# Depletion of *Numb* and *Numblike* in Murine Lung Epithelial Cells Ameliorates Bleomycin-Induced Lung Fibrosis by Inhibiting the β-Catenin Signaling Pathway

**DOI:** 10.3389/fcell.2021.639162

**Published:** 2021-05-26

**Authors:** Alessandro Ianni, Michael Hofmann, Poonam Kumari, Shahriar Tarighi, Hamza M Al-Tamari, André Görgens, Bernd Giebel, Hendrik Nolte, Marcus Krüger, Isabelle Salwig, Soni Savai Pullamsetti, Andreas Günther, André Schneider, Thomas Braun

**Affiliations:** ^1^Department of Cardiac Development and Remodeling, Max-Planck-Institute for Heart and Lung Research, Bad Nauheim, Germany; ^2^Department of Lung Development and Remodeling, Max-Planck-Institute for Heart and Lung Research, Bad Nauheim, Germany; ^3^Institute for Transfusion Medicine, University Hospital Essen, Essen, Germany; ^4^Cologne Excellence Cluster on Cellular Stress Responses in Aging-Associated Diseases (CECAD)-Cluster of Excellence, Köln, Germany; ^5^Member of the German Center for Lung Research (DZL), Member of the Cardio-Pulmonary Institute (CPI), Bad Nauheim, Germany; ^6^Universities of Giessen and Marburg Lung Center (UGMLC), Justus-Liebig-University, Giessen, Germany

**Keywords:** lung, fibrosis, epithelium, NUMB, β-catenin

## Abstract

Idiopathic pulmonary fibrosis (IPF) represents the most aggressive form of pulmonary fibrosis (PF) and is a highly debilitating disorder with a poorly understood etiology. The lung epithelium seems to play a critical role in the initiation and progression of the disease. A repeated injury of lung epithelial cells prompts type II alveolar cells to secrete pro-fibrotic cytokines, which induces differentiation of resident mesenchymal stem cells into myofibroblasts, thus promoting aberrant deposition of extracellular matrix (ECM) and formation of fibrotic lesions. Reactivation of developmental pathways such as the Wnt-β-catenin signaling cascade in lung epithelial cells plays a critical role in this process, but the underlying mechanisms are still enigmatic. Here, we demonstrate that the membrane-associated protein NUMB is required for pathological activation of β-catenin signaling in lung epithelial cells following bleomycin-induced injury. Importantly, depletion of *Numb* and *Numblike* reduces accumulation of fibrotic lesions, preserves lung functions, and increases survival rates after bleomycin treatment of mice. Mechanistically, we demonstrate that NUMB interacts with casein kinase 2 (CK2) and relies on CK2 to activate β-catenin signaling. We propose that pharmacological inhibition of NUMB signaling may represent an effective strategy for the development of novel therapeutic approaches against PF.

## Introduction

Idiopathic pulmonary fibrosis (IPF) is a progressive lung disorder characterized by increased deposition of extracellular matrix (ECM), which results in pathological changes of lung architecture and deterioration of lung functions, ultimately leading to death (Todd et al., 2012; Raghu et al., 2018). Activation of resident mesenchymal stem cells (MSCs) and subsequent differentiation into ECM-secreting myofibroblasts are assumed to be the principal causes for accumulation of fibrotic lesions (Todd et al., 2012; Camelo et al., 2014). Numerous studies indicated a critical role of lung epithelial cells, in particular alveolar epithelial cells (AECs), for the initiation and progression of IPF (reviewed in Katzen and Beers, 2020). The alveolar epithelium is mainly composed of two types of pneumocytes: AEC type I and type II (ATI and ATII cells, respectively). ATI pneumocytes are large and thin cells that cover 90–95% of the alveolar surface, mediating gas exchange. In contrast, the cuboidal ATII pneumocytes secrete antimicrobial proteins as well as the lipoprotein complex “surfactant” and promote tissue repair following injury, mainly through differentiation into ATI cells (Jansing et al., 2017). It is currently assumed that critical events in the pathogenesis of IPF include repetitive injuries of ATII cells such as chronic endoplasmic reticulum stress (Korfei et al., 2018) and subsequent accumulation of cellular dysfunctions such as loss of stemness, increased apoptosis, and induction of senescence, among others (Tanjore et al., 2011; Hill et al., 2019; Parimon et al., 2020). Additionally, dysfunctional ATII cells promote the accumulation of fibrotic lesions by paracrine signals that act as pro-fibrotic factors by stimulating proliferation and differentiation of resident MSCs and lipofibroblasts into myofibroblasts (Tanjore et al., 2011; Selman and Pardo, 2012, 2014). ATII cells may directly contribute to lung fibrosis by undergoing epithelial-to-mesenchymal transition (EMT) and inducing the expression of components of the ECM, although this possibility is still debated (Kim et al., 2006; Tanjore et al., 2009; Rock et al., 2011; Todd et al., 2012). In line with the critical role in lung fibrosis, hyperplastic ATII pneumocytes have been found in lung biopsies of patients suffering from pulmonary fibrosis (PF) (Kulkarni et al., 2016). The molecular mechanisms responsible for the aberrant activation and dysfunction of AECs still remain poorly understood, although reactivation of molecular signaling pathways involved in lung development such as Wnt-β-catenin, Sonic Hedgehog, and Notch may play a decisive role in this process (Selman et al., 2008; Aoyagi-Ikeda et al., 2011; Baarsma and Konigshoff, 2017).

The Wnt-β-catenin signaling pathway is involved in numerous cellular processes. In the absence of canonical Wnt ligands, a multiprotein destruction complex facilitates phosphorylation of serine residues in the N-terminus of cytosolic β-catenin leading to β-catenin ubiquitination and proteasomal degradation (Clevers, 2006). Binding of Wnt to Frizzled receptors prevents β-catenin phosphorylation and degradation, resulting in the accumulation of β-catenin proteins in the nucleus and the activation of down-stream genes after binding to the transcription factor T-cell factor (TCF)/lymphoid enhancer factor (LEF) (Baarsma and Konigshoff, 2017). In addition to canonical Wnt-dependent β-catenin activation, other Wnt-independent pathways stimulate β-catenin signaling (Aktary et al., 2016). The tetrameric serine/threonine protein kinase casein kinase 2 (CK2) was identified as a critical factor for β-catenin signaling. CK2 inhibits different components of the multiprotein disruption complex responsible for β-catenin degradation and indirectly promotes β-catenin phosphorylation at Ser 522, thus increasing β-catenin protein stability (Gao and Wang, 2006; Ponce et al., 2011). In addition, CK2 strengthens LEF/β-catenin interactions by direct phosphorylation of LEF resulting in stronger transactivation of target genes (Wang and Jones, 2006).

The aberrant activation of β-catenin promotes differentiation of MSCs into myofibroblasts and thus plays a critical role in lung fibrosis. Wnt-β-catenin signaling is activated in ATII cells following bleomycin-induced lung injury, a well-established experimental model of lung fibrosis (Chen et al., 2016; Baarsma and Konigshoff, 2017). The inhibition of the Wnt-β-catenin signaling pathway ameliorates bleomycin-induced PF in mice, mainly by attenuating the secretion of pro-fibrotic cytokines such as the transforming growth factor β-1 (TGF β-1) by ATII cells (Chen et al., 2016). Increased Wnt-β-catenin signaling has been observed in clinical samples derived from PF patients, which further supports the critical role of this pathway in lung fibrosis (Chilosi et al., 2003; Konigshoff et al., 2008, 2009; Baarsma and Konigshoff, 2017).

NUMB protein was originally discovered in *Drosophila melanogaster*, as a cell fate determinant in the development of bristles, peripheral sensory organs of the fly (Uemura et al., 1989). In mammals, NUMB homologs are encoded by two different genes: *Numb* and *Numblike* (*Numbl*; Gulino et al., 2010). Mouse NUMB seems to exert broader functions when compared with the *Drosophila* homolog, probably due to the presence of four different isoforms generated by alternative splicing (Dho et al., 1999; Verdi et al., 1999). NUMB contains two main domains: a N-terminal phospho-tyrosine-binding domain (PTB) and a C-terminal proline-rich domain (PRR) that are both affected by alternative splicing: the NUMB 1 and 2 splicing isoforms carry a short amino acid sequence insert within the PTB domain, which is not present in the NUMB 3 and 4 splicing isoforms. The insert mediates the association of NUMB to the plasma membrane. Hence, NUMB 1 and 2 are membrane-associated while NUMB 3 and 4 accumulate in the cytoplasm. Moreover, NUMB 1 and 3 contain a large amino acid sequence insert within the PRR domain that is not present in the other isoforms (Dho et al., 1999).

NUMB controls numerous biological processes such as endocytosis, cell adhesion, cell polarity, and migration, among others. Previous studies revealed that NUMB proteins play a critical role in the regulation of different signaling pathways including Notch, Sonic Hedgehog, and p53 (Gulino et al., 2010). *Numb* is essential for mouse development, and *Numb*-deficient mice die around embryonic day 11.5 (E11.5) due to severe neural defects. In contrast, *Numbl* knockout mice do not show any obvious developmental phenotype (Zhong et al., 2000; Petersen et al., 2002). NUMB is highly expressed in mammalian lung, but its role in PF remains largely uncharacterized. Interestingly, recent work demonstrated that NUMB promotes epithelial-to-mesenchymal transition (EMT) in response to TGF-β1 stimulation in ATII cells *in vitro*, although the underlying molecular mechanisms have not been characterized further (Zhang et al., 2018).

Here, we demonstrate that NUMB plays a critical role in the stimulation of β-catenin signaling in lung epithelial cells both *in vivo* and *in vitro*. We found that inactivation of *Numb* and *Numblike* in the lung epithelium diminishes the formation of ATII cells, reduces β-catenin signaling, and attenuates lung fibrosis following bleomycin-induced lung injury. Our experiments show that NUMB forms a molecular complex with CK2 and promotes β-catenin activation in a CK2-dependent manner.

## Materials and Methods

### Generation of Lung Epithelial Conditional NUMB Knockout Animals (*Numb/I dKO*)

*Numb*-floxed mice were provided by Zilian et al. (2001) and crossed with constitutive *Numblike* knockout (KO) animals (Petersen et al., 2004). For the generation of lung epithelial *Numb* cKO animals, we used a tetracycline inducible system (Tet-On system). *Numblike* KO/*Numb*-floxed mice were bred with transgenic animals expressing the reverse tetracycline transactivator (rtTA) under the control of the human SPC promoter and the Cre recombinase under the control of the tet operator (SPCrtTA//tetOCre; Perl et al., 2002). This system allows the expression of the Cre recombinase in the lung epithelium upon administration of doxycycline in drinking water, resulting in recombination of the *Numb*-floxed alleles. To trace Cre expression and for fluorescence-activated cell sorting (FACS), mice were crossed with Rosa26-Stopflox-EYPF reporter line (obtained from Jackson Laboratory).

### Animal Husbandry, Bleomycin Administration, and Measurement of Lung Mechanics

All animal experiments were performed in accordance with the Guide for the Care and Use of Laboratory Animals published by the National Institutes of Health and were approved by the local authorities (RP Darmstadt).

Animals were housed in individual ventilated cages with food and water provided *ad libitum* on a 12-h−based light/dark cycle. For bleomycin treatment, 10–12-week-old male mice were injected intratracheally with 2.5 U/kg bleomycin (Bleomedac^®^) dissolved in 0.9% NaCl solution. Mice were sacrificed 14 or 21 days post-injury as indicated. Control mice were treated with 0.9% NaCl as vehicle. The measurement of lung mechanics was performed using the flexiVent^TM^ system (SCIREQ^®^, Emka Technologies) as described previously (Al-Tamari et al., 2018).

### Tissue Preparation, Immunohistochemistry, and Immunofluorescence

Mice were narcotized and subjected to trans-cardiac perfusion with phosphate-buffered saline (PBS). Lungs were cannulated via the trachea, inflated with 1% PFA and fixed *in situ* for 5 min at room temperature (RT). The lungs were then ligated, dissected from the thoracic cavity, and fixed for additional 2 h in 4% PFA at 4°C. After incubation, the lungs were washed extensively in PBS and incubated with 30% sucrose at 4°C overnight. After incubation, tissues were embedded in Tissue-Tek^®^ for cryosections or in paraffin. For paraffin sections, fixed lungs were dehydrated through a series of graded ethanol baths. Samples were incubated in isopropanol for 2 h at RT and transferred to a solution of isopropanol/paraffin (1:1) at 65°C for 2 h before embedding in 100% paraffin at 65°C.

Hematoxylin and eosin (H&E) staining was performed on paraffin sections following standard procedures. Briefly, sections were deparaffinized by washing twice in xylene and rehydrated by incubation in decreasing concentrations of ethanol to ultrapure water. Sections were transferred in Mayer’s hematoxylin (Waldeck) for 10 min at RT, washed twice in tap water, dipped 10 times in 2% HCl/70% ethanol, and washed twice in double distilled water. Slides were then stained with eosin (Waldeck) for 7 min. Samples were dehydrated through a series of ethanol baths to xylene and mounted with Entellan (Merck). The degree of fibrosis was assessed according to the Ashcroft score as described previously (Ashcroft et al., 1988; Al-Tamari et al., 2018). The score scales the grade of fibrotic changes in histological lung samples numerically (from 0 to 8: 0: normal lung; 1: minimal fibrous thickening of bronchial and epithelial walls; 2–3: moderate thickening without damage of lung architecture; 4–5: fibrosis with damage of lung architecture and accumulation of fibrous masses; 6–7: severe distortion of lung structure and large fibrous areas; and 8: total fibrous obliteration of the field). At least 30 sections from each individual lung were scanned and analyzed in a single-blinded manner. Images were acquired with a Keyence BZ-9000 microscope.

For immunofluorescence staining, fixed cells or tissue cryosections were blocked and permeabilized with 1% bovine serum albumin (BSA)/5% goat serum/0.3% Triton X-100 for 15 min. After treatment, samples were incubated overnight with appropriate primary antibody diluted in 1% BSA/5% goat serum/0.1% Triton X-100 in a humidified chamber at 4°C. On the next day, samples were washed five times in PBS and incubated with fluorophore-conjugated secondary antibodies for 1 h at RT. Slides were washed three times in PBS, and cellular nuclei were counterstained using 4′,6-diamidino-2-phenylindole (DAPI). The slides were mounted with Mowiol^®^ (Millipore). Images were captured using a confocal scanner microscope (CLSM-Leica—TCS SP2). The following primary antibodies were used in this study: anti-β-catenin (Cell Signaling Technology; #9562), anti-pro-SPC (Millipore; ab3786), anti-E-cadherin (Abcam; ab11512), and anti-NUMB (Cell Signaling Technology; #2756) antibodies.

### Measurement of Collagen Content in Whole-Lung Homogenates

Collagen content in whole-lung homogenates was measured using the Sircol^TM^ Collagen Assay Kit (Biocolor Ltd.) following the manufacturer’s instructions.

### Isolation of Primary Lung Epithelial Cells and FACS

Primary lung epithelial cells were isolated as described previously (Driscoll et al., 2012). Cells were resuspended in FACS sorting buffer (0.5 M EDTA, pH 8.0, 1 M HEPES in PBS) and subjected to FACS using a BD FACSAria^TM^ III flow cytometer (BD Biosciences) equipped with a 405/488/561/633 four-laser system.

### Cell Culture and Treatment

MLE12 lung epithelial cells were purchased from ATCC and cultivated in DMEM (Sigma-Aldrich) supplemented with 10% fetal calf serum (FCS, Sigma-Aldrich), 100 U/ml penicillin, 0.1 mg/ml streptomycin, and 2 mM glutamine (Sigma-Aldrich) at 37°C in a humidified atmosphere with 5% CO_2_. For bleomycin treatment, cells were incubated with 50 μg/ml bleomycin (Bleomedac^®^) for 24 h before harvest.

### Generation of Stable Cell Lines

Stable scramble and *Numb* knockdown cells were generated using lentiviral-driven expression of shRNA as previously described (Ianni et al., 2017). In this study, the following shRNAs were used: scramble shRNA: CCTAAG GTTAAGTCGCCCTCGCTCGAGCGAGGGCGACTTAACCTT AGG-3′; *Numb* shRNA: CCGGGCTGGTTAGAAGAAG TGTCAACTCGAGTTGACACTTCTTCTAACCAGCTTTTTG. For generation of stable MLE12 cells expressing enhanced green fluorescent protein (eGFP)-tagged human NUMB 1–4, N-terminal eGFP-fused *NUMB* isoforms were cloned into the lentiviral vector pCL6-EG-wo. Lentiviral particles were generated by co-transfection of 293T HEK cells with pCL6-EG-wo vector, the helper plasmid pCD/NL-BH, and the envelope plasmid pcoPE in a ratio of 5:5:1. After transfection, lentiviral particles were harvested and used for MLE12 cell transduction as already described (Gorgens et al., 2014). eGFP-positive cells were isolated using flow cytometric cell sorting.

### RNA Extraction and Quantitative RT-PCR

Total RNA from cultivated cells or homogenized lung tissues was extracted using peqGOLD^®^ TriFast^TM^ reagent (Peqlab) following the manufacturer’s instructions. cDNA was synthetized using SuperScript^TM^ II Reverse Transcriptase kit (Invitrogen). The synthetized cDNA was diluted 1:20 in distilled water and used as template for qPCR. Quantification of the relative mRNA expression was achieved by employing the TaqMan^®^ Gene Expression Assay using a StepOnePlus Real-Time PCR System (Applied Biosystems). *Gapdh* was used as a loading control. The quantification of the relative expression of the analyzed genes was calculated using the ΔΔCt method (Livak and Schmittgen, 2001) using the StepOne^TM^ Software v2.3. The following TaqMan^®^ probes were used in this study: *Numb* (Mm01302750_m1), *Numbl* (Mm01171278_m1), *Spc* (Mm00488146_g1), *Cc10* (Mm01230908_m1), *Podoplanin* (Mm01348912_g1), *Foxj1* (Mm01267279_m1), *Ascl2* (Mm01268891_g1), *Col1a1* (Mm00801666_g1), *Ctgf* (Mm01192933_g1), *Acta2* (Mm00725412_s1), *Wisp1* (Mm01200484_m1), *Fibronectin* (Mm01256744_m1), *Snai1* (Mm00441533_g1), *Twist1* (Mm00442036_m1), *Axin1* (Mm01299060_m1), Tgf-β1 (Mm01178820_m1), and *Gapdh* (Mm99999915_g1).

### Western Blot Analysis and Co-immunoprecipitation

Protein lysates from cultivated cells or homogenized tissues were obtained by resuspending the samples in protein lysis buffer (66 mM Tris-HCl and 2% SDS) supplemented with protease and phosphatase inhibitors (0.5 μg/ml benzamidine, 2 μg/ml aprotinin, 2 μg/ml leupeptin, 2 mM PMFS, 1 mM Na_3_VO_4_, and 20 mM NaF). Protein lysates were sonicated and clarified by centrifugation. Protein concentrations were determined with the DC^TM^ Protein Assay Kit (Bio-Rad). Equal amounts of proteins were prepared in protein lysis buffer containing a final concentration of 15% glycerol, 50 mM dithiothreitol (DTT), and bromophenol blue. The samples were boiled at 95°C for 5 min and resolved by western blotting using standard procedures. The following primary antibodies were used in this study: anti-NUMB (Cell Signaling Technology; #2756), active non-phospho-β-catenin (Cell Signaling Technology; #8814), pan β-catenin (Cell Signaling Technology; #9562), Anti-Tag (CGY)FP (Evrogen; ab121), cortactin (Cell Signaling Technology; #3502), CK2α (Cell Signaling Technology; #2656), CK2β (Thermo Fisher Scientific; #PA5-27416), AKT (Cell Signaling Technology; #2967), p-AKT (Abcam: ab133458), and GAPDH (Cell Signaling Technology; 2118). The intensity of bands was quantified using Image Lab^TM^ software (version 6.0.1; Bio-Rad Laboratories) and normalized to loading controls. The normalized signal of the control sample for each experiment was set to 100. The abundance of target protein is displayed as fold change relative to controls (relative expression). Co-immunoprecipitation experiments were performed as already described (Ianni et al., 2017, 2021). The following antibodies were used in this study: anti-NUMB (Cell Signaling Technology; #2756), anti-Tag (CGY)FP (Evrogen; ab121), CK2α (Thermo Fisher Scientific; #LF-MA0223), and cortactin (Upstate; 05-180).

### Mass Spectrometry Analysis for Identification of NUMB Interactor Partners

NUMB interactor partners in MLE12 lung epithelial cells were identified using mass spectrometry analysis. Stable eGFP and NUMB-1-eGFP-overexpressing cells were subjected to co-immunoprecipitation using GFP-Trap^®^ _MA Beads (ChromoTek) following manufacturer’s instructions. The immunoprecipitates were resolved by SDS-PAGE and subjected to in gel digestion and mass spectrometry (MS) analysis. SDS-PAGE gels were stained with InstantBlue^TM^ Staining Kit (Instant Blue, Expedeon) as recommended by the manufacturer. Gel lanes were cut into 1-cm^2^ squares and collected in Eppendorf tubes. Gel pieces were destained in 50 mM ammonium bicarbonate (ABC)/50% ethanol for 20 min at room temperature and dehydrated with 100% ethanol. Proteins were reduced by incubation with 10 mM dithiothreitol (DTT) at 56°C for 45 min and then alkylated with 55 mM iodoacetamide (IAA)/50 mM ABC for 30 min. Samples were washed sequentially with the following: 50 mM ABC, 100% ethanol, 50 mM ABC, and then twice in 100% ethanol. Peptide digestion was performed with 4.6 ng/μl trypsin in 50 mM ABC overnight at 37°C. On the next day, the supernatant containing digested peptides was collected into separate tubes. Gel pieces were incubated with increasing concentrations of acetonitrile [30% acetonitrile/3% trifluoroacetic acid (TFA) for 20 min, 70% acetonitrile for 20 min, and then twice with 100% acetonitrile for 20 min]. In each step, the supernatant containing extracted peptides was collected and mixed together. Digested peptides were mixed 1:1 with a solution of 5% acetonitrile and 1% trifluoroacetic acid. Peptides were separated using a binary buffer system of buffer A [0.1% (*v*/*v*) formic acid in H_2_0] and B [0.1% (*v*/*v*) formic acid in 80% acetonitrile] on an EASY nanoflow HPLC system (Thermo Fisher Scientific, Odense Denmark) and loaded on 50-cm columns (75 μm ID) packed in-house with C18 resin (1.9 μm diameter). The HPLC was coupled to a quadrupole-based mass spectrometer Q Exactive (Thermo Fisher Scientific, Bremen, Germany) via a nanoelectrospray ionization source (Thermo Fisher Scientific, Bremen, Germany). MS spectra were acquired at a resolution of 70,000 (200 m/z) in a mass range of 350–1,650 m/z. Tandem mass spectrometry (MS/MS) events were measured in a data-dependent mode for the 10 most abundant peaks (Top10 method) in the high mass accuracy Orbitrap after HCD (higher-energy C-trap dissociation) fragmentation at 25 collision energy in a 100–1650 m/z mass range. The resolution was set to 17,500 at 200 m/z combined with an injection time of 60 ms.

Raw files were processed using MaxQuant (1.3.0.5) and the implemented Andromeda search engine. For protein assignment, electrospray ionization (ESI)-MS/MS fragmentation spectra were correlated with the UniProt mouse database (downloaded 2011), including the human NUMB protein (P49757) and a list of common contaminants. Searches were performed with tryptic digestion specificity allowing two missed cleavages and a mass tolerance of 4.5 parts per million (ppm) for MS and 6 ppm for MS/MS spectra. Carbamidomethyl at cysteine residues was set as a fixed modification, whereas oxidation at methionine and acetylation at the N-terminus were defined as variable modifications. The minimal peptide length was set to seven amino acids and the false discovery rate for proteins and peptides below 1%, using the implemented decoy algorithm. For label-free quantification, the minimum ratio was set to 2. Significantly enriched proteins were identified using a two-sided Student’s *t*-test between control (eGFP) and eGFP-NUMB1 immunoprecipitations. Targets enriched more than twofold in the eGFP-NUMB1 immunoprecipitations as compared to controls (*p*-value < 0.05) were considered as potential NUMB 1 interactor partners.

### Statistical Analysis

Data are expressed as the mean ± standard deviation (SD) of at least three independent biological replicates. Statistical significance was assessed by Student’s *t*-test or one-way ANOVA as indicated in each experiment using GraphPad Prism 5.0 software.

## Results

### Inactivation of *Numb* in MLE12 ATII Cells Attenuates Activation of β-Catenin Signaling Following Bleomycin Exposure

**FIGURE 1:**
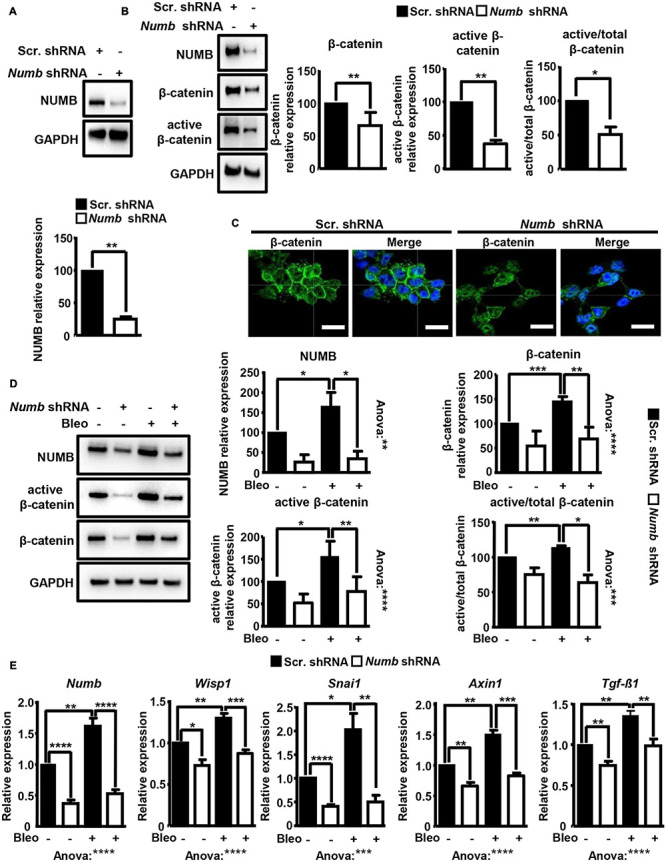
Depletion of NUMB in lung epithelial cells attenuates β-catenin signaling *in vitro.* **(A)** Western blot analysis of NUMB in MLE12 lung epithelial cells stably expressing scramble or *Numb*-targeting shRNA. GAPDH was used as a loading control. Quantification of NUMB expression ± SD is shown in the graph below (*n* = 3; **p < 0.01; Student’s *t*-test). **(B)** Western blot analysis of total and active β-catenin in scramble and *Numb* knockdown (KD) cells. GAPDH was used as a loading control. Quantification of total and active β-catenin relative to GAPDH and active/total β-catenin ± SD is shown on the right (*n* = 6 for total β-catenin and *n* = 3 for active β-catenin; *p < 0.05, **p < 0.01; Student’s *t*-test). **(C)** Immunofluorescence (IF) staining of *Numb* KD cells for total β-catenin (green). Nuclei were counterstained with DAPI (*n* = 3; scale bar = 15 μm). **(D)** Western blot analysis for total and active β-catenin in scramble and *Numb* KD cells, 24 h after treatment with bleomycin. GAPDH was used as loading control. Quantification of NUMB and of total and active β-catenin expression ± SD is shown on the right. Statistical significance between two specific groups was assessed by Student’s *t*-test. Analysis of variance between all measured groups was done using the one-way ANOVA test (*n* = 4; *p < 0.05, **p < 0.01, ***p < 0.001, ****p < 0.0001). **(E)** RT-qPCR analysis of known β-catenin target genes (*Wisp1*, *Snai1*, *Axin1*, and *Tgf-*β*1*) in scramble and *Numb* KD cells 24 h after bleomycin treatment. Efficient inhibition of *Numb* in KD cells was assessed by RT-qPCR. β-Actin was used as a loading control. Quantification of average mRNA levels relative to β-actin ± SD is shown in the histograms (*n* = 6; *p < 0.05, **p < 0.01; ***p < 0.001, and ****p < 0.0001; Student’s *t*-test and one-way ANOVA as indicated).

We were intrigued by a recent study, reporting that NUMB promotes bleomycin-induced PF through a yet unknown molecular mechanism (Zhang et al., 2018). To gain further insight into the biological function of NUMB in lung epithelial cells, we generated stable MLE12 ATII cell lines lacking NUMB expression by using a shRNA targeting *Numb*. Western blot analysis confirmed efficient inhibition of NUMB expression in this cell line (Figure 1A). Since the activation of Wnt-β-catenin signaling in lung epithelial cells plays a critical role in promoting lung fibrosis, we analyzed whether NUMB influences the expression and activation of β-catenin. Importantly, we observed a substantial reduction of total and active β-catenin levels in *Numb* knockdown (KD) cells as compared to scramble controls (Figure 1B). An immunofluorescence analysis for β-catenin confirmed these data and revealed a dramatic reduction in the amount of membrane-associated β-catenin in NUMB-depleted cells (Figure 1C). To analyze whether NUMB influences β-catenin activation following bleomycin-induced injury, we analyzed the expression of total and active β-catenin in control and *Numb* KD MLE12 cells after exposure to bleomycin for 24 h. Repression of *Numb* massively suppressed the activation of β-catenin in response to bleomycin, suggesting that NUMB enables β-catenin signaling following injury (Figure 1D). Interestingly, bleomycin treatment did not only strongly induce expression of β-catenin but also of NUMB, further arguing for a potential supportive role of NUMB in β-catenin activation (Figure 1D). To validate these results, we determined the expression of known β-catenin target genes in NUMB-depleted cells following bleomycin treatment by quantitative RT-PCR (RT-qPCR). We found that *Numb* KD cells showed substantially lower expression of several β-catenin target genes as compared with control cells, which corresponded to reduced β-catenin levels (Figure 1E). Bleomycin treatment also increased *Numb* mRNA expression, suggesting that increased levels of NUMB protein are due to enhanced transcription or RNA stabilization (Figure 1E). Taken together, our data provide compelling evidence that NUMB promotes the activation of β-catenin signaling in injured lung epithelial cells.

### Numb and *Numblike* Are Required for the Generation of Normal Numbers of ATII Cells and β-Catenin Localization in Mice

**FIGURE 2:**
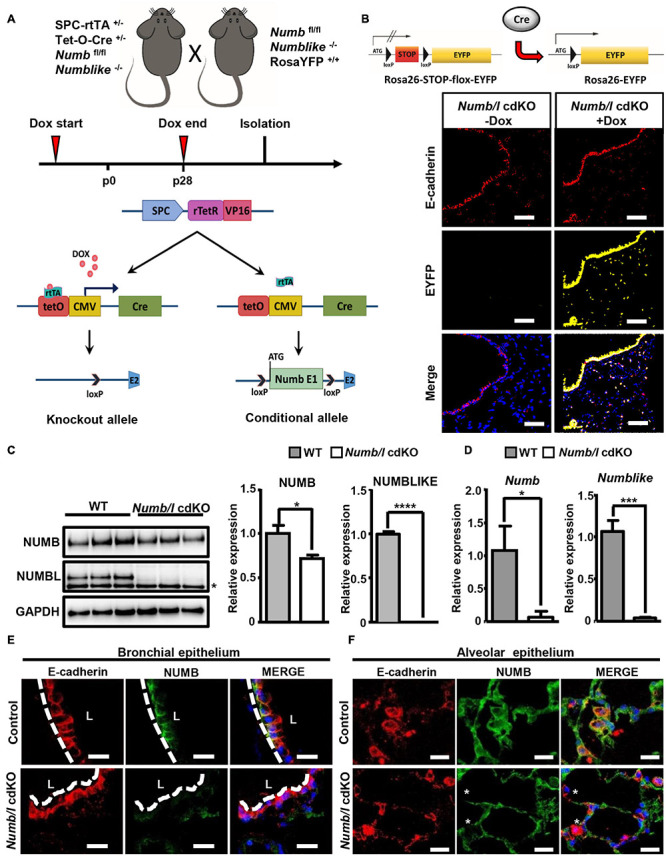
Generation and characterization of *Numb/l* cdKO animals. **(A)** Schematic model illustrating the strategy for generation of *Numb/l* cdKO animals. **(B)** A schematic representation of the Rosa26-STOP-flox-EYFP transgenic mice used to identify Cre recombinase activity in lung epithelial cells (upper panel). Immunofluorescence staining of lung cryosections from *Numb/l* cdKO animals carrying ROSA26-EGFP reporter gene without and with Dox treatment. The proximal and distal EYFP-labeled cells (yellow) and E-cadherin-positive (E-cadherin; red) lung epithelial cells are clearly visible. **(C)** Western blot analysis of NUMB and NUMBLIKE expression in whole-lung homogenates derived from WT and *Numb/l* cdKO animals. GAPDH was used as a loading control. The asterisk indicates a non-specific band. Quantification of NUMB and NUMBLIKE relative to GAPDH ± SD is shown on the right (*n* = 3; *p < 0.05, ****p < 0.0001; Student’s *t*-test). **(D)** RT-qPCR analysis of *Numb* (left panel) and *Numblike* (right panel) expression in FACS-sorted lung epithelial cells derived from WT and *Numb/l* cdKO mice. The graph represents the average relative expression ± SD of the indicated gene of three independent animals (*n* = 3; *p < 0.05, ***p < 0.001; Student’s *t*-test). **(E,F)** Immunofluorescence staining for NUMB (green) and E-cadherin (red) of lung sections derived from control and *Numb/l* cdKO adult mice (8 weeks old). Efficient depletion of NUMB in the bronchial **(E)** and alveolar epithelium **(F)** is clearly discernible. Asterisks indicate NUMB-depleted E-cadherin-positive cells. Scale bars = 12 μm.

Next, we investigated whether NUMB exerts similar functions in lung epithelial cells in mice. Since global inactivation of *Numb* in mice results in embryonic lethality (Zhong et al., 2000), we generated lung epithelial conditional *Numb* knockout animals (*Numb* cKO) using a tetracycline-inducible system (Figure 2A). We generated mice (*Numb* cKO) carrying a floxed *Numb* allele (*Numb*^*fl/fl*^), which expresses the tetracycline transactivator (rtTA) under control of the lung epithelial specific promoter Spc (SPCrtTA) (Perl et al., 2002) and the Cre recombinase under the control of the tet operator (SPCrtTA//tetOCre). The doxycycline (Dox)-dependent expression of Cre recombinase allows specific deletion of *Numb* in lung epithelial cells (Figure 2A). To avoid possible compensatory effects by the *Numb* homolog *Numblike*, we additionally introduced a *Numblike* knockout allele into *Numb* cKO animals, which was feasible since *Numblike* germ line mutants do not show any obvious developmental phenotype (Petersen et al., 2002). We also added a *Rosa26-EYFP* allele to the SPCrtTA//tetOCre//*Numb*^*fl/fl*^//*Numblike^–/–^* strain, which expresses EYFP after Dox-mediated activation of Cre recombinase expression and subsequent recombination, to enable tracing of recombined cells *in vivo* (Figure 2B). Hereafter, we refer to SPCrtTA//tetOCre// *Numb*^*fl/fl*^//*Numblike^–/–^*//*Rosa26-EYFP* mice as *Numb/l* cdKO and *Numb*^*fl/fl*^//*Numblike^–/–^*//*Rosa26-EYFP* as control mice. To ensure complete recombination of the *Numb*^*fl/fl*^ alleles, *Numb/l* cdKO mice received Dox via the drinking water throughout gestation until postnatal stage p28 (Wert et al., 1993; Perl et al., 2002). Immunofluorescence staining of lung sections from *Numb/l* cdKO mice for EYFP verified that the activity of the Cre recombinase was restricted to the alveolar and bronchiolar lung epithelium (Figure 2B). No EYFP signal was detected in control *Numb/l* cdKO mice, which did not receive Dox treatment, confirming the validity of our model.

**FIGURE 3:**
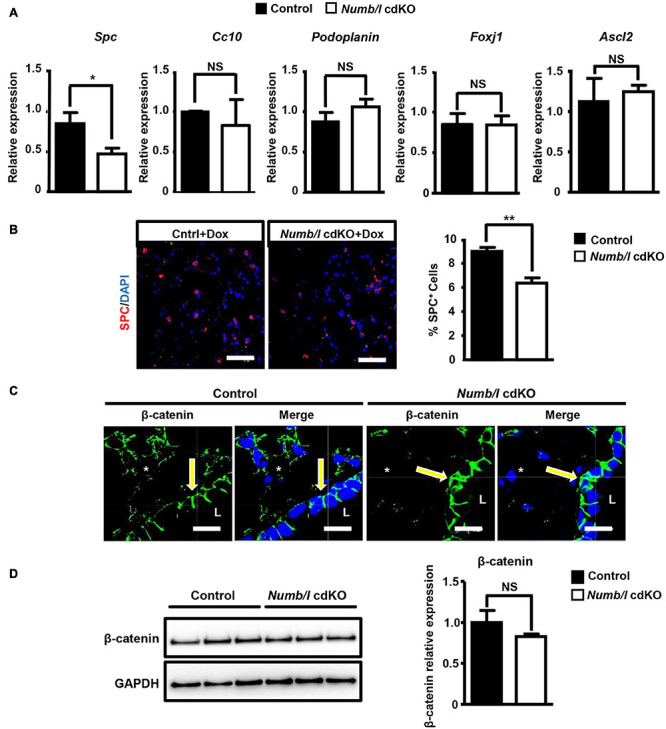
*Numb/l* are required for generation of normal numbers of ATII cells in the mouse lung and normal localization of β-catenin in lung epithelial cells. **(A)** RT-qPCR analysis of lung epithelial cell markers (*Spc*: ATII cells; *Cc10*: Club cells; *Podoplanin*: ATI cells; *Foxj1*: ciliated cells, and *Ascl2*: neuroendocrine cells) in total lung homogenates derived from control and *Numb/l* cdKO mice (*n* = 3, *p < 0.05; NS, not significant). **(B)** Immunofluorescence staining for SPC of lung sections derived from control and *Numb/l* cdKO animals. Cell nuclei were counterstained with DAPI. Scale bar = 47 μm. Quantification of SPC-positive cells is shown in the histogram on the right (*n* = 3 per group; **p < 0.01). **(C)** Immunofluorescence staining for β-catenin of lung sections derived from control and *Numb/l* cdKO animals. Cell nuclei were counterstained with DAPI. Scale bar = 12 μm. Notice the aberrant localization of β-catenin in bronchial epithelial cells (arrows) and the decline in β-catenin expression in alveolar cells (asterisks). **(D)** Western blot analysis of β-catenin in whole-lung homogenates derived from control and *Numb/l* cdKO animals. GAPDH was used as a loading control. Quantification of β-catenin expression ± SD is shown in the graph on the right (*n* = 4; NS, not significant; Student’s *t*-test).

In order to demonstrate efficient inactivation of *Numb* and *Numblike* (*Numbl*) in knockout mice, we performed a RT-TaqMan^®^ gene expression analysis. As expected, cdKO mice showed a complete absence of *Numbl* expression. *Numb* mRNA levels were reduced in total lung homogenates, although the difference did not reach significance (Supplementary Figure 1A). In contrast, western blot analysis demonstrated a significant reduction of NUMB and NUMBL proteins in cdKO animals compared to controls (Figure 2C). We assume that the expression of *Numb* outside epithelial cells obscures depletion in the epithelium. Therefore, we took advantage of the EYFP reporter to specifically isolate lung epithelial cells by fluorescence-activated cell sorting (FACS; Supplementary Figure 1B). As expected, we measured a virtually complete absence of *Numb* and *Numbl* expression in FACS-isolated EYFP^+^-cells from cdKO animals (Figure 2D). To further confirm efficient depletion of NUMB in the lung epithelium, we performed co-staining of NUMB and the epithelial marker E-cadherin by immunofluorescence on lung sections derived from control and *Numb/l* cdKO Rosa26-EYFP*^–/–^* embryos (Supplementary Figure 1C). As expected, we observed a co-localization of NUMB with E-cadherin in the lung epithelium of control animals, which was absent in *Numb/l* cdKO mice (Supplementary Figure 1C). Similar results were obtained in adult animals, where NUMB was detected in E-cadherin-positive bronchial and alveolar epithelial cells of wild-type but not *Numb/l* cdKO animals (Figures 2E,F, respectively). *Numb/l* cdKO mice were born at Mendelian ratio and did not show any gross morphological abnormalities. However, a more refined analysis revealed a substantial reduction of mRNA expression of the well-established ATII cell marker *Spc* in *Numb/l* cdKO mice compared to control animals, whereas the expression of the Club cell marker *Cc10*, the ATI cell marker *Podoplanin*, the ciliated cell marker *Foxj1*, and the neuroendocrine cell marker *Ascl2* was not changed (Figure 3A). In line with this finding, an immunofluorescence analysis of pro-SPC-positive cells followed by a morphometric analysis revealed a reduction of ATII cells of approximately 30% in *Numb/l* cdKO mice as compared to control littermates (Figure 3B). We concluded that NUMB is required for the generation of regular numbers of ATII cells in the lung epithelium but not for their proper positioning or normal lung morphogenesis. The normal expression of Club, ATI, neuroendocrine, and ciliated cell markers but not of ATII markers strongly suggested that only ATII but no other epithelial cells were reduced in *Numb/l* cdKO mice, although such an extrapolation has to be viewed with some caution.

To investigate whether the absence of NUMB has an impact on the distribution and expression of β-catenin in lung epithelial cells *in vivo*, we stained lung sections from adult *Numb/l* cdKO mice for β-catenin. Strikingly, we observed a profound reduction of β-catenin in the ATII cell-containing alveolar epithelium and a shift of β-catenin from a mostly lateral to a basolateral position in *Numb* mutant bronchial epithelial cells (Figure 3C). In contrast, western blot analysis did not reveal a significant reduction of β-catenin levels in whole-lung lysates from *Numb/l* cdKO mice (Figure 3D), since the presence of other cell types expressing high levels of β-catenin in the lung obscured the reduction of β-catenin levels in ATII cells. Taken together, our results clearly indicate that the absence of *Numb/l* in lung epithelial cells leads to a severe disorganization of β-catenin in bronchial cells and a dramatic reduction of β-catenin expression in alveolar epithelial cells.

### NUMB-Dependent Activation of β-Catenin Signaling Requires CK2

**FIGURE 4:**
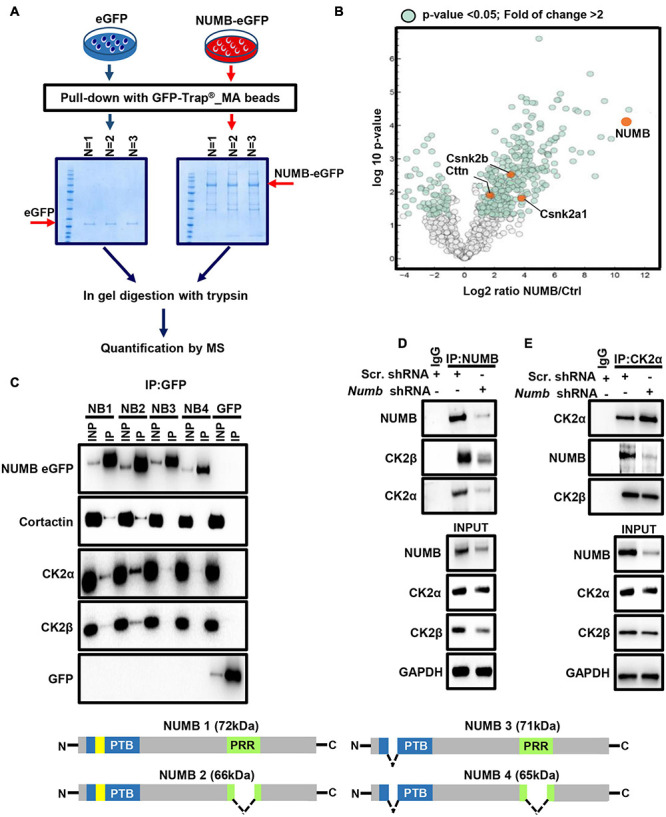
NUMB interacts with different proteins including CK2. **(A)** A scheme depicting the experimental approach used for mass spectrometry-based analysis of NUMB–eGFP interactor partners. **(B)** A volcano plot showing NUMB-interacting proteins detected by mass spectrometry. **(C)** Coupled immunoprecipitation (GFP antibody) and western blot analysis (GFP, cortactin, CK2α, and CK2β antibodies) of stable NUMB 1–4-overexpressing cells (*n* = 3; upper panel). The lower scheme illustrates the structure of the four NUMB isoforms (PTB, phospho-tyrosine-binding domain; PRR, proline-rich domain; yellow box, amino acid insert in the PTB domain) that might be responsible for interaction with specific targets. See text for details. **(D)** Coupled immunoprecipitation (NUMB antibody) and western blot analysis (NUMB, CK2α, and CK2β antibodies) of scramble and *Numb* KD cells. Non-immune immunoglobulin (IgG) was used as a negative control (*n* = 3). **(E)** Coupled immunoprecipitation (CK2α antibody) and western blot analysis (NUMB and CK2β antibodies) of scramble and *Numb* KD cells.

To investigate the molecular mechanism governing NUMB-mediated stimulation of β-catenin signaling, we searched for potential novel NUMB interaction partners (Figure 4A). Stable MLE12 cells expressing eGFP-tagged NUMB were subjected to immunoprecipitation using GFP-Trap^®^ _MA beads, and co-precipitated proteins were analyzed by mass spectrometry (Figure 4A). In line with already described functions of NUMB (Gulino et al., 2010), we found that NUMB interacts with several proteins involved in cellular vesicle assembly, cell–cell contact formation, and other cellular functions (Figure 4B, Supplementary Figure 2A, and Supplementary Table 1). In addition, we identified several novel interaction partners, including cortactin (cttn) and the CK2α (Csnk2a1) and β subunits (Csnk2b) (Figure 4B and Supplementary Table 1). To validate the mass spectrometry results, we performed co-immunoprecipitation experiments in MLE12 cells stably expressing different NUMB isoforms (NUMB 1–4) fused to eGFP (Figure 4C). Cortactin and CK2α and β robustly co-precipitated with both NUMB 1 and NUMB 2 while negligible interactions were detected with the other NUMB isoforms (Figure 4C). These results suggest that the amino acid insert in the PTB domain, which is only present in the NUMB 1 and 2 isoforms, is critical for the interaction with cortactin and CK2α and β (Figure 4C). In addition, we found that NUMB 1 co-localizes with cortactin, while depletion of NUMB in MLE12 cells caused aberrant sub-cellular localization of cortactin (Supplementary Figures 2B,C). Since CK2 plays a critical role in β-catenin activation (Gao and Wang, 2006; Ponce et al., 2011), we further characterized the interaction between NUMB and CK2. Co-immunoprecipitation experiments using scramble and *Numb* shRNA-treated MLE12 cells revealed that not only the transfected NUMB–eGFP fusion proteins but also endogenous NUMB formed a molecular complex with CK2 (Figures 4D,E). Interestingly, quantification of CK2 protein levels demonstrated that depletion of *Numb* in lung epithelial cells significantly reduced the expression of CK2β while the α subunit remained unchanged, indicating that NUMB not only interacts with CK2β but also controls its expression (Supplementary Figure 2D).

**FIGURE 5:**
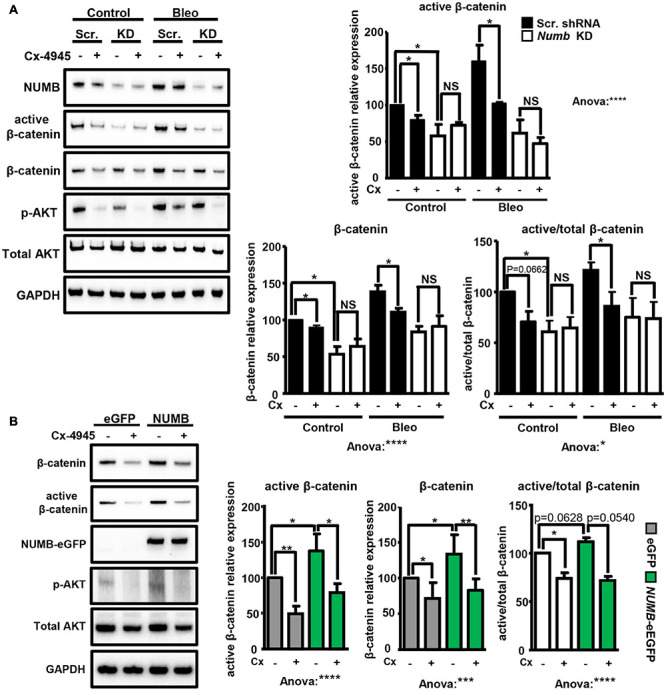
NUMB-dependent activation of β-catenin requires CK2 activity. **(A)** Western blot analysis of the levels of indicated markers in scramble (Scr.) and *Numb* KD MLE12 cells 24 h after bleomycin treatment in the presence of 10 μM CK2 inhibitor Cx-4945 as indicated. Phosphorylated AKT at serine 129 (p-AKT) was used as a positive control to demonstrate efficient inhibition of CK2. GADPH was used as loading control. Quantification of total and active β-catenin relative levels ± SD is given in the histograms below (*n* = 4; *p < 0.05, ****p < 0.0001; NS, not significant; Student’s *t*-test and one-way ANOVA as indicated). **(B)** Western blot analysis of active and total β-catenin expression levels in stable e-GFP-tagged NUMB-overexpressing cells in the presence of Cx-4945 inhibitor for 24 h. GAPDH was used as a loading control. Quantification of active and total β-catenin relative levels ± SD is given in the histograms below (*n* = 4; *p < 0.05, **p < 0.01, Student’s *t*-test; One-way ANOVA: ***p < 0.001, ****p < 0.0001).

Next, we investigated whether NUMB-dependent stimulation of β-catenin signaling requires CK2 activity. Exposure of scramble and *Numb* shRNA-treated cells to bleomycin in the presence or absence of the CK2 specific inhibitor Cx-4945 revealed that Cx-4945 efficiently reduced the levels of active and total β-catenin in scramble control but not in NUMB-depleted cells (Figure 5A). An analysis of AKT phosphorylation at serine 129 (p-AKT), a major target of CK2, confirmed efficient inhibition of CK2 activity by Cx-4945 (Figure 5A; Ponce et al., 2011). Taken together, these data clearly indicate that the presence of NUMB is necessary for CK2-dependent regulation of β-catenin signaling. Furthermore, we analyzed the expression of total and active β-catenin in stable NUMB-overexpressing cells in the presence of the CK2 inhibitor Cx-4945. Pharmacological inhibition of CK2 prevented NUMB-mediated stimulation of β-catenin signaling, corroborating the role of CK2 in NUMB-dependent stimulation of β-catenin (Figure 5B).

### Loss of *Numb/l* Ameliorates Bleomycin-Induced Lung Fibrosis *in vivo*

**FIGURE 6:**
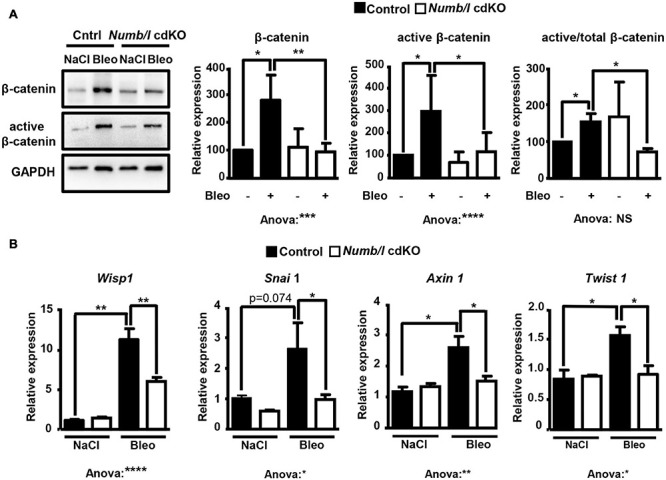
Depletion of NUMB in lung epithelial cells attenuates β-catenin signaling *in vivo* following bleomycin injury. **(A)** Western blot analysis of total and active β-catenin in lung homogenates from control and *Numb/l* cdKO animals. GAPDH was used as a loading control. Quantification of total and active β-catenin expression ± SD is shown on the right (*n* = 5; *p < 0.05, **p < 0.01, ***p < 0.001, ****p < 0.0001; Student’s *t*-test and one-way ANOVA as indicated). **(B)** RT-qPCR analysis of β-catenin target genes in lung homogenates from control and *Numb/l* cdKO animals 14 days after treatment with vehicle (NaCl) or bleomycin (Bleo) (*n* = 3, *p < 0.05, **p < 0.01, ****p < 0.0001).

**FIGURE 7:**
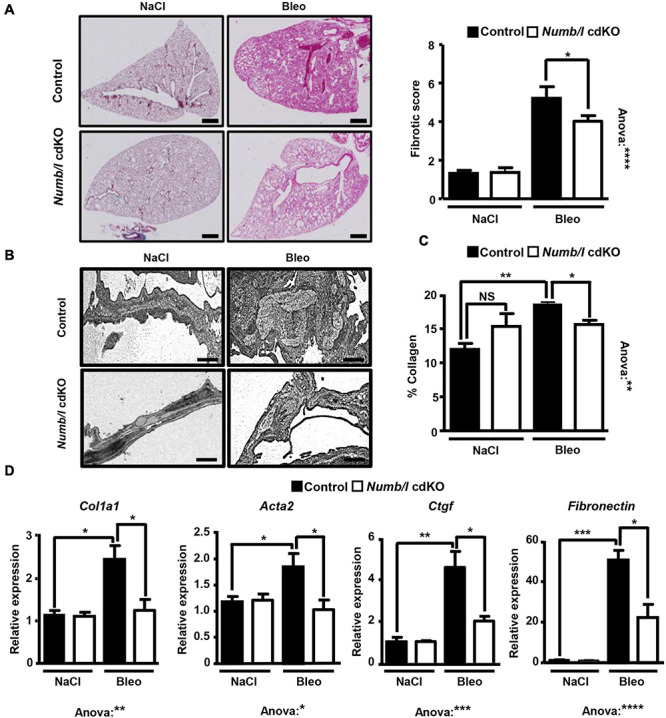
Inactivation of *Numb/l* in lung epithelial cells ameliorates bleomycin-induced lung fibrosis. **(A)** H&E staining of paraffin sections (5 μm) from *Numb/l* cdKO vs. control mice 21 days after bleomycin treatment. Scale bar = 1 mm. Quantification of the fibrotic foci is on the right. The fibrotic score was assigned arbitrarily between 0 (non-fibrotic) and 8 (highly fibrotic) using randomly chosen H&E-stained slides from control and *Numb/l* cdKO mice (control: *n* = 3; *Numb/l* cdKO: *n* = 5; *p < 0.05, ****p < 0.0001). **(B)** Electron microscopy analysis of collagen I fiber deposition in control and *Numb/l* cdKO animals 21 days after treatment with vehicle (NaCl) or bleomycin (Bleo). Scale bar = 1 μm. **(C)** Sircol-based analysis of collagen content in whole-lung homogenates derived from control and *Numb/l* cdKO animals (*n* = 4; *p < 0.05, **p < 0.01; NS, not significant). **(D)** RT-qPCR analysis of pro-fibrotic genes in lung homogenates from control and *Numb/l* cdKO animals (control: *n* = 3; *Numb/l* cdKO: *n* = 5; *p < 0.05, **p < 0.01, ***p < 0.001, ****p < 0.0001).

To analyze whether attenuation of β-catenin signaling in *Numb/l* cdKO mice protects against PF, we injected control and mutant mice with bleomycin and determined the expression of total and active β-catenin in whole-lung homogenates 14 days after injury. In line with previous results (Li et al., 2015; Chen et al., 2016; Cao et al., 2018), control mice showed a strong increase of total and active β-catenin after bleomycin injection (Figure 6A). In sharp contrast, *Numb/l* cdKO mice displayed negligible β-catenin activation, indicating a critical role for NUMB in the activation of β-catenin signaling *in vivo* (Figure 6A). To validate the inhibition of β-catenin signaling, we determined the expression of typical β-catenin targets, such as *Wisp1*, *Snai1*, *Axin1*, and *Twist1* 14 days after bleomycin treatment. We found that the absence of *Numb/l* strongly attenuated the upregulation of β-catenin targets compared to control animals (Figure 6B). Since we assumed that accumulation of fibrotic tissue might take longer than the activation of β-catenin, we examined lung sections 7 days later, at day 21 after bleomycin injury by H&E staining to examine the consequences of enhanced β-catenin signaling. Sections were scored for fibrosis according to the Ashcroft method (see “Materials and Methods”), revealing a substantial reduction of fibrosis in *Numb/l* cdKO compared to control animals (Figure 7A). Likewise, electron microscopy and Sircol analysis indicated reduced deposition of collagen fibers in *Numb/l* cdKO mice compared to control animals (Figures 7B,C). To further confirm these findings, we analyzed the expression of genes linked to fibrosis, such as collagen 1 (*Col1a1*), smooth muscle actin (*Acta2*), connective tissue growth factor (*Ctgf*), and *fibronectin* in whole-lung homogenates from control and *Numb/l* cdKO animals 14 days after exposure to bleomycin. In contrast to control littermates, no significant increase was spotted in *Numb/l* cdKO mice providing compelling evidence that depletion of NUMB prevents fibrosis after bleomycin injury (Figure 7D).

**FIGURE 8:**
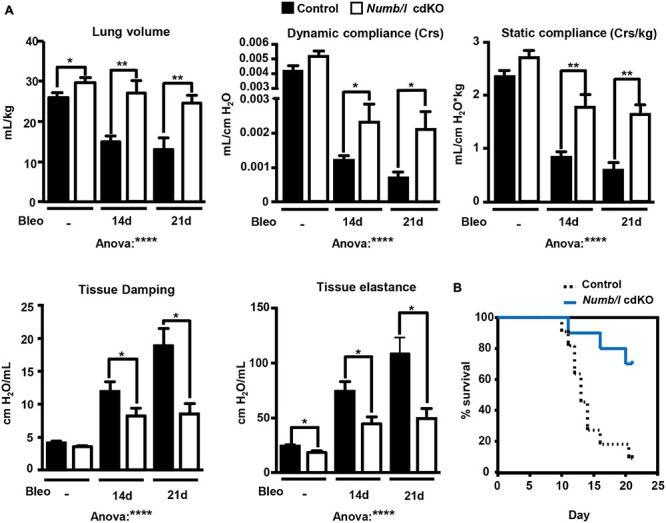
Suppression of *Numb/l* expression improves lung function and survival after bleomycin treatment. **(A)** Assessment of physiological lung parameters in control and *Numb/l* cdKO animals 14 and 21 days after treatment with vehicle (NaCl) or bleomycin (Bleo). Histograms represent the average ± SD of individual parameters (control with NaCl: *n* = 8; *Numb/l* cdKO with NaCl: *n* = 7; control with bleomycin 14 days: *n* = 10; *Numb/l* cdKO with bleomycin 14 days: *n* = 6; control with bleomycin 21 days: *n* = 3; *Numb/l* cdKO with bleomycin 21 days: *n* = 5; *p < 0.05; **p < 0.01, ****p < 0.0001). **(B)** Survival curves of control (*n* = 11) and *Numb/l* cdKO (*n* = 10) animals after bleomycin treatment.

An improvement in morphology might not necessarily correspond to improvement in lung function. Therefore, we measured several functional lung parameters in mechanically ventilated control and *Numb/l* cdKO animals 14 and 21 days after bleomycin treatment, including volume, static and dynamic compliance, tissue damping, and elastance. Compliance is defined as the change in lung volume that occurs per unit upon change in pressure and therefore provides a direct readout for the elasticity of the lung. Further information is obtained by measurement of static and dynamic compliance, which are determined in the absence of airflow and during rhythmic breathing, respectively. Importantly, depletion of *Numb* prevented a decline in lung volume and maintained higher static and dynamic compliance following bleomycin injury (Figure 8A). In addition, we measured lung damping and elastance. Lung damping is a parameter that directly correlates with the energy dissipated by the alveoli, whereas elastance, the reciprocal value of compliance, represents the variation in pressure required to change a unit of volume. Both parameters reflect tissue resistance and inversely correlate with lung elasticity. We found that *Numb/l* mutant mice showed reduced tissue dampening and strongly improved elasticity compared to control mice following injury (Figure 8A). Even more striking, inactivation of *Numb/l* dramatically increased survival after bleomycin treatment. Nearly 80% of *Numb/l* cdKO animals were still alive 20 days after the injury, compared to approximately 20% in the control group (Figure 8B). Taken together, our data indicate that inactivation of *Numb/l* in lung epithelial cells greatly ameliorates bleomycin-induced lung fibrosis, most likely by attenuating CK2-dependent β-catenin activation.

## Discussion

IPF is a progressive disease with a poorly understood etiology. Despite evident signs of increased inflammation, augmented oxidative stress, and aberrant function of the coagulation system, therapeutic approaches based on antioxidants, immunosuppressants, and/or anticoagulants failed to delay the progression of IPF (Demedts et al., 2005; Staitieh et al., 2015; Adegunsoye and Strek, 2016; Magnini et al., 2017). In patients with IPF, the use of the two antifibrotic drugs nintedanib and pirfenidone slows down the decline of lung function and improves outcome, but does not completely stop further deterioration (Korfei et al., 2018). The therapeutic effect of nintedanib and pirfenidone has been recently also proven in other forms of PF, different from IPF, which share the progressive fibrotic phenotype (Maher et al., 2018; Distler et al., 2019; Flaherty et al., 2019). Still, lung transplantation is the only strategy to treat severely ill patients, but prognosis remains poor after transplantation (Thabut et al., 2009). Thus, a better understanding of the molecular mechanisms driving the pathogenesis of IPF is imperative for the development of novel therapeutic strategies.

Interactions between epithelial cells (especially ATII cells), fibroblasts, and immune cells play a critical role for PF pathogenesis (Parimon et al., 2020). It is widely assumed that repetitive and prolonged damage of lung epithelial cells caused by viral infections, tobacco smoking, and aging causes epithelial cell dysfunction, leading to activation of pro-fibrotic signaling pathways that ultimately promote the accumulation of fibrotic lesions (Parimon et al., 2020). The activation of Wnt-β-catenin signaling is tightly linked to PF. Numerous studies document hyperactivation of the pathway in experimental models of PF as well as in human patients (Konigshoff et al., 2008; Chen et al., 2016; Baarsma and Konigshoff, 2017). Consistent with these studies, pharmacological inhibition of Wnt-β-catenin attenuates fibrosis in mice (Baarsma and Konigshoff, 2017). It has been reasoned that Wnt-β-catenin signaling contributes to PF at different levels. β-catenin-dependent signals stimulate proliferation and migration of resident fibroblasts (Lam et al., 2011) and promote differentiation of resident mesenchymal stem cells into myofibroblasts (Li et al., 2015; Cao et al., 2018). The activation of Wnt-β-catenin signaling in ATII cells leads to secretion of factors that act on resident mesenchymal stem cells to induce differentiation into myofibroblasts (Wynn, 2011; Aumiller et al., 2013; Chen et al., 2016). In addition, Wnt-β-catenin stimulates EMT in ATII cells, thus enhancing the expression of components of the ECM responsible for accumulation of fibrotic lesions (Konigshoff et al., 2009; Mutze et al., 2015; Liu et al., 2019).

Despite these insights, the mechanisms responsible for hyperactivation of β-catenin signaling in lung epithelial cells remain largely unexplored (Konigshoff et al., 2008). Here, we demonstrate that the membrane-associated protein NUMB plays a pivotal role in the regulation of β-catenin signaling in lung epithelial cells. Inactivation of *Numb/l* in the lung epithelium prevents activation of β-catenin and attenuates fibrosis, therefore preserving lung functions following bleomycin-induced injury. We reason that NUMB-dependent activation of β-catenin signaling in lung epithelial cells may promote fibrosis by enhanced secretion of cytokines such as *Wisp1*. Consistent with our data, a recent study demonstrated that NUMB is upregulated in fibrotic kidney *in vivo* where it promotes tissue fibrosis by stimulating EMT of renal epithelial cells (Ding et al., 2015). However, NUMB-mediated control of the β-catenin signaling is apparently complex and cell type-specific. In contrast to its role in fibrotic tissues, NUMB inhibits EMT in breast and ovarian cancer, partially by suppressing β-catenin signaling (Hu et al., 2019; Liang et al., 2019). Such opposing effects in different tissues or cells emphasize a high level of complexity exerted by NUMB in control of the β-catenin pathway that warrants further investigation.

The exact molecular mechanisms by which NUMB activates β-catenin signaling in lung epithelial cells require further experiments. Our data demonstrate that NUMB promotes β-catenin activation *in vitro* through interaction with CK2, a key molecule involved in β-catenin stabilization and transcriptional activation (Gao and Wang, 2006; Ponce et al., 2011). We hypothesize that binding of NUMB to CK2 might stimulate its enzymatic activity or modulate CK2 interaction with other proteins, controlling the activation of β-catenin signaling. Moreover, depletion of *Numb* decreases CK2β expression in lung epithelial cells, indicating that NUMB might promote CKβ stabilization either by direct or indirect mechanisms. Although our study clearly establishes a role of CK2 in NUMB-dependent control of β-catenin activation, additional studies are clearly required to gain better insights into the underlying molecular mechanisms. Nevertheless, our conclusion is supported by recent studies, which demonstrate critical pro-fibrotic functions of CK2 in the skin and liver (Zhang et al., 2015; Huang et al., 2017), suggesting that NUMB might promote tissue fibrosis by activating the CK2–β-catenin axis. However, it is possible that NUMB controls different signaling cascades in addition to CK2, which might indirectly activate the β-catenin pathway. For example, NUMB may either stimulate or inhibit the Notch pathway, depending on the cellular context (McGill and McGlade, 2003; McGill et al., 2009; Luo et al., 2020). Notch signaling, on the other hand, either inhibits or stimulates the Wnt-β-catenin pathway, raising the possibility that NUMB controls β-catenin activation at least in part via Notch (Hayward et al., 2005; Kwon et al., 2011; Ishiguro et al., 2017). Finally, NUMB may control the activation of β-catenin signaling by interfering with its interaction with E-cadherin. This idea is supported by previous studies demonstrating that NUMB and its homolog NUMBL interact with E-cadherin and regulate its subcellular localization (Kuo et al., 2006; Rasin et al., 2007). E-cadherin and β-catenin are both important components for the maintenance of adherens junction (Harris and Tepass, 2010). It has been proposed that E-cadherin might act as an inhibitor of β-catenin signaling by sequestering β-catenin protein, although the fraction of β-catenin involved in cell–cell contact formation might differ from the fraction involved in Wnt signaling (Hulsken et al., 1994). Decreased E-cadherin–β-catenin interactions seem to activate β-catenin signaling and lead to its translocation into the nucleus (Chilosi et al., 2003). NUMB may disable the interaction between E-cadherin and β-catenin and thus contribute to β-catenin activation, although future studies are required to substantiate such a model.

Inactivation of *Numb/l* in mouse lung epithelia did not only prevent fibrosis after bleomycin treatment but also had a clear effect on the generation of normal numbers of ATII cells during development. It seems likely that attenuation of Wnt-β-catenin signaling due to the absence of NUMB is the main cause for reduced ATII cell formation, since the Wnt-β-catenin pathway plays a pivotal role for ATII cell expansion during lung alveologenesis and maturation (Frank et al., 2016). The reduced numbers of ATII cells may also contribute to the protection against fibrosis by diminishing release of pro-fibrotic factors from fewer ATII cells or by other reasons. Finally, NUMB may exert additional functions in ATII cells, which might play a role for survival under bleomycin treatment, since the dramatic improvement in survival of *Numb/l* cdKO animal was more pronounced than expected from the reduction in fibrosis. Such mechanisms might synergize with the blunted activation of the β-catenin signaling in *Numb/l* cdKO mice.

## Conclusion

In conclusion, we demonstrate that *Numb/l* play a critical role for allowing regular expansion of ATII cells during lung development and for promoting lung fibrosis. Our data indicate that *Numb/l* are required for the activation of β-catenin signaling in lung epithelial cells following injury, probably by stimulating CK2, thereby leading to stabilization and enhanced transcriptional activation of β-catenin. We assume that pharmacological approaches targeting the NUMB/CK2/β-catenin axis represent a promising strategy to develop innovative therapies for the treatment of PF.

## Data Availability Statement

The raw data supporting the conclusions of this article will be made available by the authors, without undue reservation.

## Ethics Statement

The animal study was reviewed and approved by the Regierungspräsidium Darmstadt.

## Author Contributions

TB and AS designed all experiments. MH and AI performed the majority of the experiments with the contribution from PK, ST, HA-T, HN, MK, and IS performed data analysiing and interpreted data. AGö and BG provided stable eGFP-NUMB1-4 overexpressing MLE12 cells. TB and AI wrote the manuscript. SP and AGü helped in design the experiments and contributed to writing and editing the manuscript. TB and AS supervised the project. All authors read the manuscript and provided critical comments.

## Conflict of Interest

The authors declare that the research was conducted in the absence of any commercial or financial relationships that could be construed as a potential conflict of interest.

## Supplementary Material

Supplementary Figure 1 Characterization of *Numb/l* cdKO animals. **(A)** RT-qPCR analysis of *Numb* (left) and *Numblike* (right) expression in whole-lung homogenates from WT and *Numb/l* cdKO animals. Histograms show the average ± SD of relative mRNA expression of three different mice for each group (*n* = 3; **p* < 0.05; NS, not significant). **(B)** Lung epithelial cells were isolated from control and *Numb/l* cdKO animals and subjected to FACS sorting for isolation of EYFP-positive cells. Wild-type mice (Rosa26-EYFP^–/–^) were used as a negative control. Dead cells were identified by DAPI staining (P1). Viable cells (outside of P1) were analyzed for YFP fluorescence (fluorescein; FITC) to establish the gates for YFP^+^-positive cells. A representative experiment is shown. **(C)** Immunofluorescence staining for NUMB (green) and E-cadherin (red) on lung cryosections from control and *Numb/l* cdKO E16.5 embryos. Note the co-localization of NUMB with the epithelial marker E-cadherin and its specific depletion in *Numb/l* cdKO mice (*n* = 3; scale bar = 15 μm).

Supplementary Figure 2 NUMB interacts with cortactin. **(A)** Gene ontology (GO) enrichment analysis of potential NUMB interactor partners. **(B)** IF staining for GFP and cortactin of stable NUMB-eGFP-overexpressing cells. Nuclei were counterstained with DAPI (scale bar = 12 μm). **(C)** IF staining for cortactin of stable scramble (Scr. shRNA) and *Numb* KD cells (*Numb* shRNA). Nuclei were counterstained with DAPI (scale bar = 12 μm). **(D)** Quantification of CK2α and β levels relative to GAPDH ± SD in inputs of the co-immunoprecipitation experiments shown in Figures 4D,E (*n* = 5; **p* < 0.05; NS, not significant).

Supplementary Table 1 List of NUMB interaction partners. Excel list containing all potential interaction partners of NUMB, as detected by mass spectrometry analysis of co-IPs of eGFP-tagged NUMB in MLE12 cells.
