# Supplementary figures and images for: Depletion of Numb and Numblike in Murine Lung Epithelial Cells Ameliorates Bleomycin-Induced Lung Fibrosis by Inhibiting the β-Catenin Signaling Pathway
Source: Front Cell Dev Biol. 2021 May 26;9:639162. doi: 10.3389/fcell.2021.639162 (PMC8187792; doi:10.3389/fcell.2021.639162)

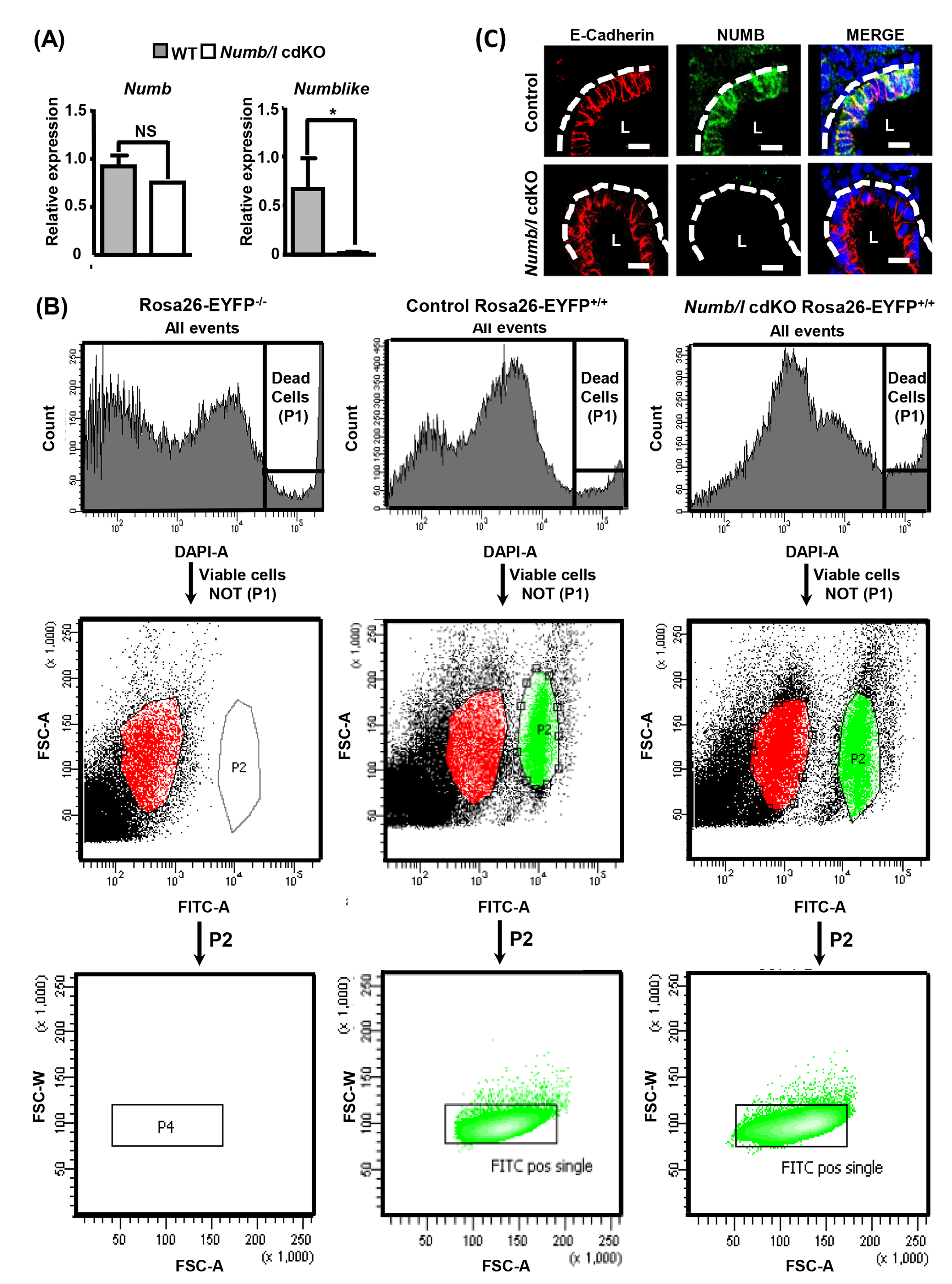

Supplement: Supplementary Figure 1 — Characterization of Numb/l cdKO animals. (A) RT-qPCR analysis of Numb (left) and Numblike (right) expression in whole-lung homogenates from WT and Numb/l cdKO animals. Histograms show the average ± SD of relative mRNA expression of three different mice for each group (n = 3; *p < 0.05; NS, not significant). (B) Lung epithelial cells were isolated from control and Numb/l cdKO animals and subjected to FACS sorting for isolation of EYFP-positive cells. Wild-type mice (Rosa26-EYFP–/–) were used as a negative control. Dead cells were identified by DAPI staining (P1). Viable cells (outside of P1) were analyzed for YFP fluorescence (fluorescein; FITC) to establish the gates for YFP+-positive cells. A representative experiment is shown. (C) Immunofluorescence staining for NUMB (green) and E-cadherin (red) on lung cryosections from control and Numb/l cdKO E16.5 embryos. Note the co-localization of NUMB with the epithelial marker E-cadherin and its specific depletion in Numb/l cdKO mice (n = 3; scale bar = 15 μm). [file Image_1.TIF]

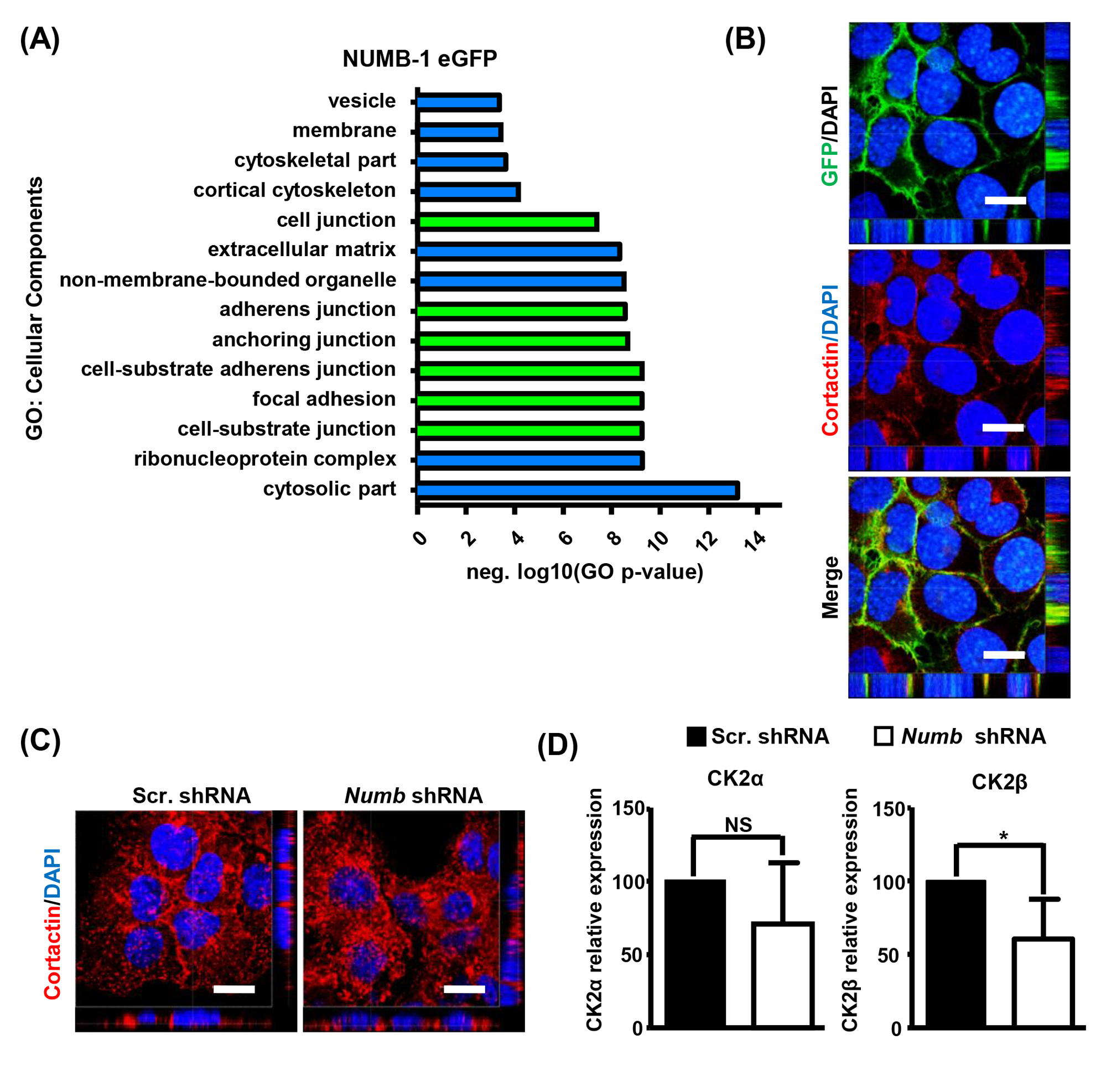

Supplement: Supplementary Figure 2 — NUMB interacts with cortactin. (A) Gene ontology (GO) enrichment analysis of potential NUMB interactor partners. (B) IF staining for GFP and cortactin of stable NUMB-eGFP-overexpressing cells. Nuclei were counterstained with DAPI (scale bar = 12 μm). (C) IF staining for cortactin of stable scramble (Scr. shRNA) and Numb KD cells (Numb shRNA). Nuclei were counterstained with DAPI (scale bar = 12 μm). (D) Quantification of CK2α and β levels relative to GAPDH ± SD in inputs of the co-immunoprecipitation experiments shown in Figures 4D,E (n = 5; *p < 0.05; NS, not significant). [file Image_2.TIF]
